# A prognostic analysis of patients with acute leukemia and a history of central nervous system involvement undergoing umbilical cord blood transplantation

**DOI:** 10.1097/BS9.0000000000000237

**Published:** 2025-06-11

**Authors:** Zichang Shen, Bingbing Yan, Xinyue Zhang, Yue Wu, Xiaoyu Zhu

**Affiliations:** a Department of Hematology, The First Affiliated Hospital of USTC, Division of Life Sciences and Medicine, University of Science and Technology of China, Hefei, Anhui 230001, China; b Anhui Provincial Key Laboratory of Blood Research and Applications, Hefei, China; c Blood and Cell Therapy Institute, Division of Life Sciences and Medicine, University of Science and Technology of China, Hefei, China

**Keywords:** Acute leukemia, Central nervous system leukemia, Umbilical cord blood transplantation

## Abstract

To investigate the efficacy and prognosis of umbilical cord blood transplantation (UCBT) in patients with acute leukemia (AL) and a history of central nervous system leukemia (CNSL). We retrospectively evaluated 62 patients with AL and a history of CNSL who underwent UCBT at our center between January 2015 and December 2022 (CNSL-positive group). From a concurrent cohort (n = 777) without a history of CNSL, propensity score matching at a 1:2 ratio was conducted based on age, sex, and diagnosis to select 124 matched controls (CNSL-negative group). The results revealed a significantly higher 4-year cumulative incidence of relapse (28.8% vs 16.8%, *p* = 0.038) and central nervous system (CNS) relapse after UCBT (14.7% vs 3.7%, *p* = 0.004) in the CNSL-positive group than in the CNSL-negative group. Furthermore, the 4-year leukemia-free survival (42.0% vs 66.2%, *p* = 0.004) and overall survival (49.6% vs 69.9%, *p* = 0.009) rates in the CNSL-positive group were significantly lower than those in the CNSL-negative group. Fine–Gray proportional hazard regression multivariate analysis identified pretransplant CNSL history as an independent high-risk factor for CNS relapse after UCBT (hazard ratio [HR] = 5.710, 95% confidence interval [CI] = 1.737–18.770, *p* = 0.004). These findings underscore the need to optimize conditioning regimens and graft-vs-host disease prophylaxis and explore novel prophylactic strategies for UCBT to improve long-term survival in patients with AL and a history of CNS involvement.

## 1. INTRODUCTION

The central nervous system (CNS) is the most common site of extramedullary infiltration in acute leukemia (AL), and CNS involvement may occur at any stage of leukemia.^1^ At initial diagnosis, CNS involvement is observed in 4% to 7% of patients with acute lymphoblastic leukemia (ALL)^2,3^ vs approximately 1% to 3% in patients with acute myeloid leukemia.^4,5^ Irrespective of the disease subtype, central nervous system leukemia (CNSL) poses significant therapeutic challenges due to the blood–brain barrier (BBB), which restricts chemotherapy penetration.^6^ Despite achieving chemotherapy-induced remission, 30% to 40% of patients experience CNS relapse,^7,8^ particularly those with high-risk features (eg, adverse cytogenetics or hyperleukocytosis).^9^ Moreover, CNSL correlates with neurotoxic complications and increased secondary marrow relapse rates, ultimately compromising long-term survival outcomes in patients.^8^

Allogeneic hematopoietic stem cell transplantation (allo-HSCT) is considered an effective treatment for AL and its relapse, especially in patients at high risk of cytogenetic and molecular genetic abnormalities.^10^ Over the past few decades, umbilical cord blood transplantation (UCBT) has been widely performed in clinics. This procedure has shown unique advantages, including no harm to mothers and donors, less stringent human leukocyte antigen (HLA) matching requirements, reduced incidence and severity of chronic graft-vs-host disease (GVHD), and a potentially enhanced graft-vs-leukemia (GVL) effect.^11^

However, there are limited studies on UCBT in patients with AL and a history of CNSL at home and abroad. A prior small-sample retrospective study conducted at our center reported promising preliminary outcomes in 15 patients with AL and a history of CNSL receiving single-unit UCBT with myeloablative conditioning (MAC) regimen without antithymocyte globulin (ATG).^12^ Building on this foundation, we further expanded the cohort to assess the prognosis in patients with AL and a history of CNSL undergoing UCBT and evaluate potential risk factors for CNS relapse after UCBT.

## 2. MATERIALS AND METHODS

### 2.1. Patients

Between January 2015 and December 2022, we retrospectively evaluated 62 patients with AL and a history of CNSL before transplantation who underwent UCBT at the Department of Hematology of the First Affiliated Hospital of the University of Science and Technology of China (Anhui Provincial Hospital) (referred to as the CNSL-positive group). Among 777 patients with AL without a history of CNSL who underwent UCBT during the same period, propensity score matching at a 1:2 ratio was conducted based on age, sex, and diagnosis to select 124 matched controls (referred to as the CNSL-negative group).

This study was approved by the Ethics Committee of the First Affiliated Hospital of the University of Science and Technology of China (2025-RE-007). Informed consent was obtained from each patient or guardian prior to transplantation in accordance with the Declaration of Helsinki.

### 2.2. Transplantations

Strategies for HLA typing and cord blood selection were as reported,^12^ and all patients received single-unit cord blood obtained from the Chinese Cord Blood Bank.

For GVHD prophylaxis, all patients were administered a combination of cyclosporine (CsA) and mycophenolate mofetil (MMF).

### 2.3. Diagnosis and treatment of CNSL

Cerebrospinal fluid (CSF) examination post-transplant is not routinely performed at our center. However, patients presenting with neurological signs or symptoms (eg, headache, nausea, or vomiting) undergo immediate cranial computed tomography (CT) or magnetic resonance imaging (MRI) to rule out meningeal disease, chloroma, or CNS bleeding. If no mass, lesion, or hemorrhage was detected, patients with platelet counts ≥20 × 10^9^/L would undergo diagnostic lumbar puncture for CNSL confirmation.^13^ For confirmed cases, a triple intrathecal chemotherapy (IT) regimen consisting of methotrexate (15 mg/m²), cytarabine (40 mg/m²), and dexamethasone (10 mg) was administered twice weekly. Upon achieving cytological clearance (defined as the absence of abnormal cells in the CSF), consolidation therapy was initiated every 2 to 4 weeks, along with cranial irradiation (total dose: 18 Gy).

### 2.4. Definitions

Neutrophil engraftment was defined as the first of three consecutive days during which the neutrophil count was ≥0.5 × 10^9^/L. Meanwhile, platelet engraftment was defined as the first day when the platelet count was ≥20 × 10^9^/L without transfusion support for seven consecutive days. CNSL was defined by positive CSF cytology (the presence of at least 5 leukocytes per microliter of CSF with leukemic blast cells) or the presence of characteristic signs and symptoms of CNS or MRI or CT scans that indicated the presence of meningeal or brain infiltration.^14,15^ Isolated CNS relapse was defined as CNS relapse without any other sites of leukemia recurrence.^16^ Hematological relapse was defined as the reappearance of leukemia cells in the peripheral blood or an observation of >5% blasts in the bone marrow aspirate.^17^ Acute and chronic GVHD, cytomegalovirus infection, and hemorrhagic cystitis were diagnosed and classified according to previously published criteria.^18–21^ Survival time was defined as the time from UCBT to the latest follow-up or death.

### 2.5. Statistical analysis

Patients’ baseline characteristics were compared using Student *t* test for continuous variables and the χ^2^ test for categorical variables. Cumulative incidence curves were constructed in a competing risk setting, with death considered a competing event, to calculate the cumulative incidence of relapse (CIR) and were compared using Gray’s test. The probabilities of overall survival (OS), GVHD-free, relapse-free survival (GRFS), and leukemia-free survival (LFS) were calculated using the Kaplan–Meier method and compared using log-rank tests. To explore the independent predictors of CNSL after UCBT, univariate and multivariate analyses were performed using Fine–Gray proportional hazard regression, and grade III–IV aGVHD was treated as a time-dependent covariate. Factors with a *p* value <0.10 in the univariate analysis were included in the multivariate analysis, and a *p* value ≤0.05 indicated a significant difference. Statistical analyses were performed using EZR version 1.61 (Microsoft Corporation, Redmond, Washington) and R software (version 4.2.0).

## 3. RESULTS

### 3.1. Patient characteristics

The baseline characteristics of the CNSL-positive (n = 62) and CNSL-negative (n = 124) groups are shown in Table 1. No significant differences were observed in sex, age, weight, disease type, donor-to-recipient sex, disease status at transplantation, HLA compatibility, anti-HLA antibodies, and infused total nucleated and CD34^+^ cells between the 2 groups. In the CNSL-positive group, 8 (12.9%) patients exhibited persistent CNS involvement refractory to IT therapy, while the remaining patients attained complete remission through pretransplant IT.

**Table 1:** Characteristics of patients between CNSL-positive group and CNSL-negative group.

Characteristics	CNSL-positive group (n = 62)	CNSL-negative group (n = 124)	*p* value
Sex, n (%)			
Female	24 (38.7)	44 (35.5)	0.747
Male	38 (61.3)	80 (64.5)	
Age (y), median (range)	17.0 (0.0, 58.0)	17.0 (0.0, 57.0)	0.969
Weight (kg), median (range)	52.0 (7.0, 102.0)	50.0 (8.0, 128.0)	0.940
Diagnosis, n (%)			
AML	24 (38.7)	48 (38.7)	0.770
ALL	36 (58.1)	74 (59.7)	
Others	2 (3.2)	2 (1.6)	
Donor-to-recipient sex, n (%)			
Female to female	44 (71.0)	90 (72.6)	0.863
Others	18 (29.0)	34 (27.4)	
ABO incompatibility, n (%)			
Identical	24 (38.7)	41 (33.1)	0.808
Major incompatibility	11 (17.7)	28 (22.6)	
Minor incompatibility	18 (29.0)	39 (31.5)	
Bidirectional incompatibility	9 (14.5)	16 (12.9)	
Disease status, n (%)			
CR	48 (77.4)	109 (87.9)	0.085
PR/NR	14 (22.6)	15 (12.1)	
HLA compatibility (/10), n (%)			
≥9	4 (6.5)	20 (16.1)	0.125
8 or 7	41 (66.1)	67 (54.0)	
≤6	17 (27.4)	37 (29.8)	
AntiHLA antibodies, n (%)			
Positive	42 (67.7)	82 (66.1)	0.248
Negative	14 (22.6)	37 (29.8)	
Missing	6 (9.7)	5 (4.0)	
Infused TNCs, median (range) (×10^7^/kg)	3.1 (0.5, 14.6)	3.3 (0.5, 15.0)	0.705
Infused CD34^+^ cells, median (range) (×10^5^/kg)	2.4 (0.3, 7.7)	2.2 (0.2, 12.0)	0.270

ALL = acute lymphoblastic leukemia, AML = acute myeloid leukemia, CNSL = central nervous system leukemia, CR = complete remission, HLA = human leukocyte antigen, PR/NR = partial remission/no remission, TNCs = total nucleated cells.

### 3.2. Transplantation outcomes

**Figure 1.**
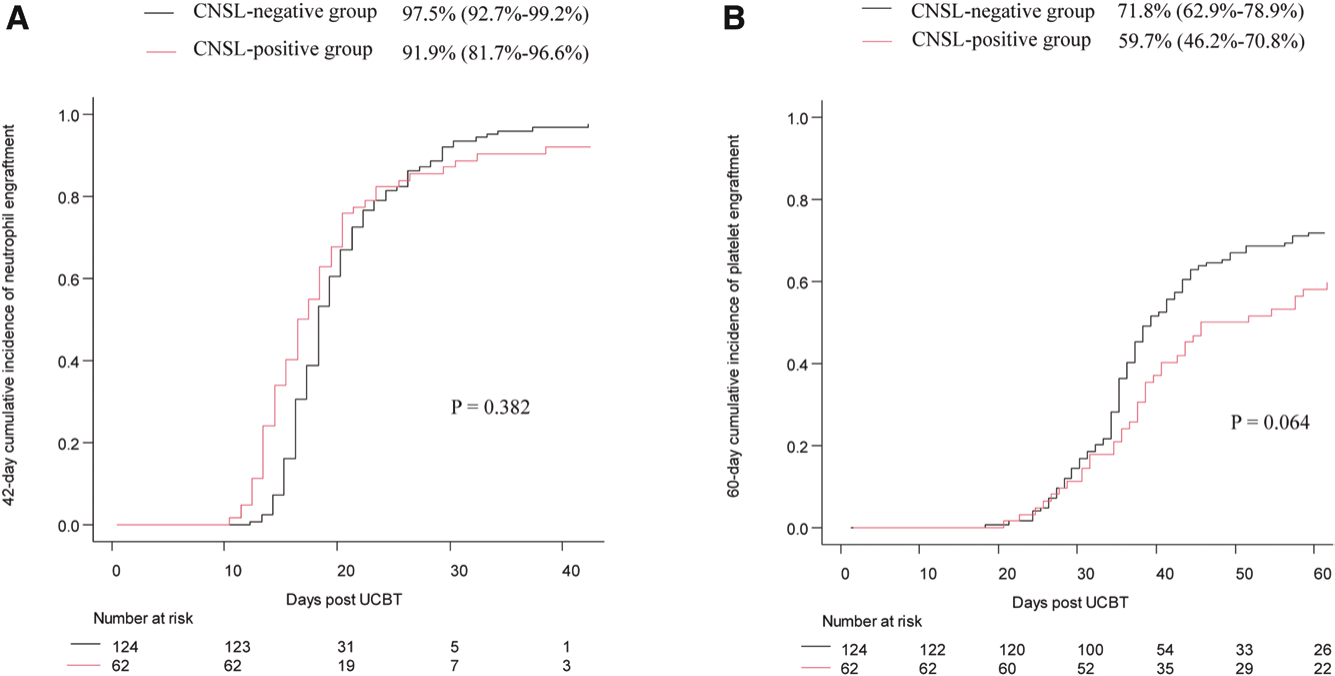
The 42-d cumulative incidence of neutrophil engraftment (A) and the 60-d cumulative incidence of platelet engraftment (B) in CNSL-positive group and CNSL-negative group. CNSL = central nervous system leukemia, UCBT = umbilical cord blood transplantation.

**Figure 2.**
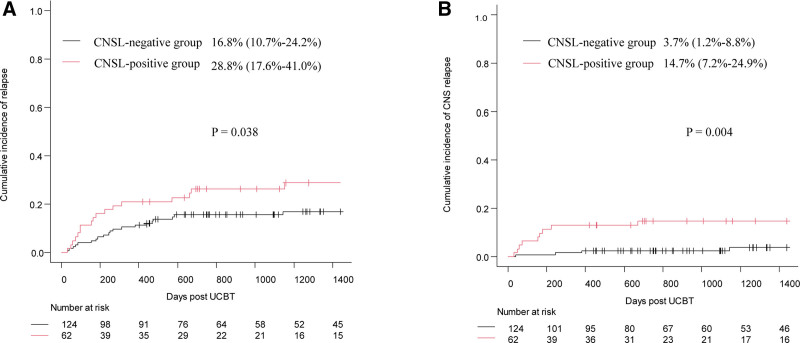
The 4-y cumulative incidence of relapse (A) and CNS relapse post-transplantation (B) in CNSL-positive group and CNSL-negative group. CNS = central nervous system, UCBT = umbilical cord blood transplantation.

**Figure 3.**
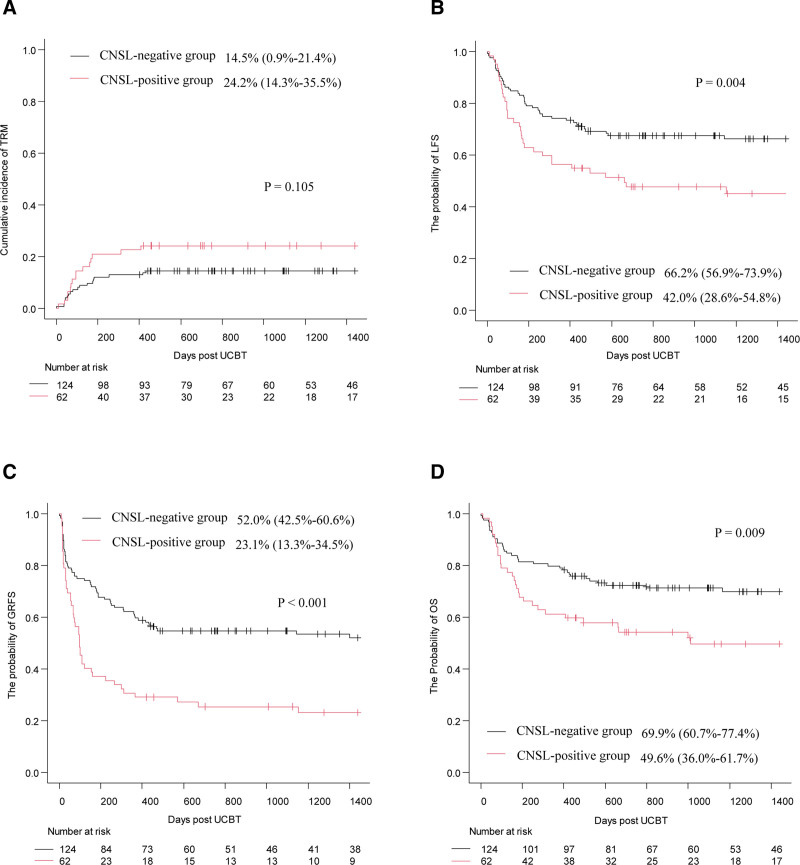
The 4-y cumulative incidence of TRM (A) and the probability of 4-y LFS (B), GRFS (C) and OS (D) in CNSL-positive group and CNSL-negative group. CNSL = central nervous system leukemia, GVHD = graft-versus-host disease, GRFS = GVHD-free relapse-free survival, LFS = leukemia-free survival, OS = overall survival, TRM = transplantation-related mortality.

All surviving patients were followed until January 31, 2025; the median follow-up times were 1766.5 days (range, 420.0–3575.0 days) in the CNSL-positive group and 1645.0 days (range, 401.0–3389.0 days) in the CNSL-negative group. Post-transplantation outcomes are presented in Table 2. The median times for neutrophil and platelet engraftment were similar between the 2 groups (both *p* > 0.05). No statistical differences were observed between 2 groups in the 42-day cumulative incidence of neutrophil engraftment (91.9% [95% confidence interval {CI}, 81.7%–96.6%] vs 97.5% [95% CI, 92.7%–99.2%], *p* = 0.382, **Fig. 1A**) and the 60-day cumulative incidence of platelet engraftment (59.7% [95% CI, 46.2%–70.8%] vs 71.8% [95% CI, 62.9%–78.9%], *p* = 0.064, **Fig. 1B**). Other transplant-related outcomes, such as the incidence of PES, acute GVHD, chronic GVHD, and hemorrhagic cystitis, did not differ between the 2 groups (Table 2). The 4-year CIR and 4-year CNS relapse were significantly higher in the CNSL-positive group than in the CNSL-negative group (CIR: 28.8% [95% CI, 17.6%–41.0%] vs 16.8% [95% CI, 10.7%–24.2%], *p* = 0.038; CNS relapse: 14.7% [95% CI, 7.2%–24.9%] vs 3.7% [95% CI, 1.2%–8.8%], *p* = 0.004, **Fig. 2A and B**). However, no significant differences were observed in the 4-year cumulative transplant-related mortality between the 2 groups (24.2% [95% CI, 14.3%–35.5%] vs 14.5% [95% CI, 0.9%–21.4%], *p* = 0.105, **Fig. 3A**). The probability of 4-year LFS, GRFS, and OS in the CNSL-positive group were significantly lower than those in the CNSL-negative group (LFS: 42.0% [95% CI, 28.6%–54.8%] vs 66.2% [95% CI, 56.9%–73.9%], *p* = 0.004; GRFS: 23.1% [95% CI, 13.3%–34.5%] vs 52.0% [95% CI, 42.5%–60.6%], *p* < 0.001; OS: 49.6% [95% CI, 36.0%–61.7%] vs 69.9% [95% CI, 60.7%–77.4%], *p* = 0.009, **Fig. 3B–D**).

**Table 2:** Transplantation outcomes between CNSL-positive group and CNSL-negative group.

Outcomes	CNSL-positive group	CNSL-negative group	*p* value
	(n = 62)	(n = 124)	
42-d cumulative incidence of neutrophil engraftment (95% CI)	91.9% (81.7%–96.6%)	97.5% (92.7%–99.2%)	0.382
60-d cumulative incidence of platelet engraftment (95% CI)	59.7% (46.2%–70.8%)	71.8% (62.9%–78.9%)	0.064
Neutrophil engraftment in days, median (range)	16.0 (10.0, 40.0)	16.0 (10.0, 38.0)	0.529
Platelet engraftment in days, median (range)	37.5 (19.0, 171.0)	36.0 (17.0, 210.0)	0.255
CMV infection, n (%)	35 (56.5)	84 (67.7)	0.147
HC, n (%)	14 (22.6)	34 (27.4)	0.594
Bacterial BSI, n (%)	18 (29.0)	34 (27.4)	0.863
100-d cumulative incidence of Grade II–IV aGVHD, (95% CI)	38.7% (26.6%–50.7%)	33.9% (25.7%–42.2%)	0.456
100-d cumulative incidence of Grade III–IV aGVHD, (95% CI)	29.0% (18.3%–40.7%)	16.1% (10.3%–23.2%)	0.040
4-y cumulative incidence of cGVHD, (95% CI)	22.6% (13.1%–33.7%)	14.0% (8.2%–21.2%)	0.082
4-y CIR, (95% CI)	28.8% (17.6%–41.0%)	16.8% (10.7%–24.2%)	0.038
4-y CNS relapse post-transplantation, (95% CI)	14.7% (7.2%–24.9%)	3.7% (1.2%–8.8%)	0.004
4-y TRM, (95% CI)	24.2% (14.3%–35.5%)	14.5% (0.9%–21.4%)	0.105
4-y LFS, (95% CI)	42.0% (28.6%–54.8%)	66.2% (56.9%–73.9%)	0.004
4-y GRFS, (95% CI)	23.1% (13.3%–34.5%)	52.0% (42.5%–60.6%)	<0.001
4-y OS, (95% CI)	49.6% (36.0%–61.7%)	69.9% (60.7%–77.4%)	0.009

aGVHD = acute graft-versus-host disease, BSI = bloodstream infection, cGVHD = chronic graft-versus-host disease, CI = confidence interval, CIR = cumulative incidence of relapse, CMV = cytomegalovirus, CNS = central nervous system, CNSL = central nervous system leukemia, GRFS = GVHD-free relapse-free survival, HC = hemorrhagic cystitis, LFS = leukemia-free survival, OS = overall survival, TRM = transplantation-related mortality.

### 3.3. Risk factors of CNS relapse after UCBT

We analyzed clinical factors that may affect CNS relapse following UCBT in the general population (n = 186) (Table 3). Univariate analyses indicated that CNSL relapse might be associated with the following factors: ALL as the underlying disease (hazard ratio [HR] = 8.978, 95% CI = 1.204–66.920, *p* = 0.032), partial remission/no remission (PR/NR) status before transplantation (HR = 3.841, 95% CI = 1.259–11.720, *p* = 0.018), infused CD34^+^ cell count (HR = 0.180, 95% CI = 0.039–0.820, *p* = 0.072), and a history of CNSL before UCBT (HR = 4.825, 95% CI = 1.505–15.470, *p* = 0.001). We further included all the possible variables above (*p* < 0.10) in the multivariate analysis, and the results revealed that ALL (HR = 14.770, 95% CI = 2.059–106.000, *p* = 0.007), PR/NR status (HR = 2.724, 95% CI *=* 1.970–10.470, *p* = 0.024), and history of CNSL (HR = 5.710, 95% CI = 1.737–18.770, *p =* 0.004) remained independent risk factors for CNS relapse after UCBT (Table 3).

**Table 3:** Univariate and multivariate analysis of the factors associated with CNS relapse post-UCBT.

Variables	Univariate analysis		Multivariate analysis	
	HR (95% CI)	*p* value	HR (95% CI)	*p* value
Age (y)				
≤17				
>17	0.687 (0.225–2.100)	0.510		
Sex				
Male				
Female	0.510 (0.142–1.829)	0.3		
Weight (kg)				
≤51				
>51	1.224 (0.412–3.635)	0.720		
Diagnosis				
AML				
ALL	8.978 (1.204–66.920)	0.032	14.770 (2.059–106.000)	0.007
Disease status				
CR				
PR/NR	3.841 (1.259–11.720)	0.018	2.724 (1.970–10.470)	0.024
ABO incompatibility				
Identical				
Major incompatibility	0.926 (0.250–3.426)	0.910		
Minor incompatibility	0.667 (0.129–3.435)	0.630		
Bidirectional incompatibility	0.521 (0.061–4.414)	0.550		
HLA compatibility (/10)				
≥9				
8 or 7	1.145 (0.131–9.979)	0.9		
≤6	2.723 (0.323–22.950)	0.360		
Infused TNCs (10^7^/kg)				
≤3.20				
>3.20	0.831 (0.643–1.072)	0.150		
Infused CD34^+^ cells (10^5^/kg)				
≤2.25				
>2.25	0.180 (0.039–0.820)	0.072	0.526 (0.322–3.859)	0.100
A history of CNSL (yes/no)	4.825 (1.505–15.470)	0.001	5.710 (1.737–18.770)	0.004
Platelet engraftment (yes/no)	0.458 (0.139–1.517)	0.2		
Grade III–IV aGVHD (yes/no)	1.412 (0.673–2.964)	0.360		
CMV infection (yes/no)	1.743 (0.476–6.384)	0.4		
Bacterial BSI (yes/no)	0.893 (0.214–3.311)	0.870		
HC (yes/no)	1.030 (0.281–3.774)	0.960		
AntiHLA antibodies (positive/negative)	0.210 (0.027–1.608)	0.130		

aGVHD = acute graft-versus-host disease, ALL = acute lymphoblastic leukemia, AML = acute myeloid leukemia, BSI = bloodstream infection, CI = confidence interval, CMV = cytomegalovirus, CNS = central nervous system, CNSL = central nervous system leukemia, CR = complete remission, HC = hemorrhagic cystitis, HLA = human leukocyte antigen, HR = hazard ratio, PR/NR = partial remission/no remission, TNCs = total nucleated cells, UCBT = umbilical cord blood transplantation.

### 3.4. Outcomes on CNS relapse following UCBT

Among the 13 patients experiencing post-transplant CNS relapse, isolated CNS involvement occurred in 6 cases (46.2%), while 7 patients (53.8%) demonstrated concurrent CNS and hematologic relapse. Among the isolated CNS relapse patients (n = 6), one patient with a critically compromised physical condition did not receive IT and died within 1 month, and the remaining patients achieved complete CSF blast clearance following triple IT regimens.

Among the patients with concurrent hematologic relapse (n = 7), 2 elected to discontinue treatment and succumbed within 30 days of relapse. The remaining 5 all received chemotherapy combined with triple IT, while 2 patients failed to achieve hematologic remission and died.

## 4. DISCUSSION

Allo-HSCT is the most effective therapeutic approach for AL, with long-term disease-free survival in recipients primarily attributable to GVL effects. Despite the advancements in allo-HSCT techniques that have reduced transplant-related mortality, primary disease relapse remains the predominant cause of post-transplant mortality, particularly in high-risk populations. Notably, prior studies have identified pretransplant CNSL history as an independent risk factor for post-transplant relapse.^22,23^ In agreement with these findings, our study demonstrates that patients with a history of CNSL exhibit significantly higher risks of systemic and CNS relapse, resulting in inferior transplantation outcomes, including LFS, GRFS, and OS.

UCBT demonstrates a potent GVL effect, especially in patients positive for minimal residual disease. Therefore, recipients have a lower relapse rate following UCBT than after unrelated donor transplantation.^24^ To synergistically improve UCB hematopoietic reconstitution with minimal residual disease eradication, our center has implemented an ATG-free intensified MAC regimen, integrating high-dose Ara-C or Fludarabine into traditional “total body irradiation/cyclophosphamide” or “busulfan/cyclophosphamide” platforms, complemented by ATG-free GVHD prophylaxis to preserve GVL potency. Furthermore, incorporating carmustine (BCNU), a BBB-penetrating alkylating agent, into the conditioning regimen of patients with CNSL significantly reduced post-transplant CNS relapse rates.

Our preliminary retrospective study showed that the CIR of 15 patients with AL and a history of CNSL undergoing UCBT was 20.00% (95% CI, 17.70%–22.30%), with 3-year OS and LFS rates of 60.0% and 53.3%, respectively.^12^ While these outcomes demonstrated therapeutic potential, the absence of controlled comparisons limited definitive conclusions. Therefore, we expanded our cohort and conducted comparative analyses with recipients without CNSL, which showed that the relapse rate of patients with a history of CNSL remained significantly higher than that of patients without. A history of CNSL has emerged as an independent risk factor for leukemia relapse and CNSL recurrence, likely mediated by the attenuated GVL effect within the immune-privileged microenvironment of the CNS.

There is no consensus regarding routine CNSL prophylaxis after allo-HSCT. In patients without prior CNS involvement, the European Society for Blood and Marrow Transplantation does not recommend routine IT prophylaxis before or after allo-HSCT.^25,26^ Meanwhile, for patients with prior CNS involvement, Liu et al^27^ found that prophylactic IT shows promise in mitigating CNS relapse rates, which still requires further prospective studies to substantiate. In addition, prophylactic IT after transplantation is associated with an increased risk of CNS complications, particularly leukoencephalopathy. In our center, post-UCBT CNS prophylaxis is not routinely performed. Our results demonstrated that the CNSL-positive group exhibited an increased CNS relapse rate, comparable to reported rates after haploidentical donor transplantation (14.7% vs 19.8%) and significantly lower than that following HLA-identical sibling donor HSCT (14.7% vs 70.0%), further demonstrating the potent GVL effect of UCBT.^27^

Our study has some limitations. First, this was a single-center retrospective analysis with a relatively small number of patients. Second, the disease risk index of patients was not included in the analysis because of missing data on chromosomes and genetic tests.

In summary, although UCBT is associated with a potent GVL effect, patients with AL and a history of CNSL have a higher risk of relapse following UCBT, along with inferior LFS, GRFS, and OS. To improve outcomes in this high-risk population, optimization of the UCBT conditioning regimen and GVHD prophylaxis should be prioritized, along with the development of novel prophylactic interventions targeting CNS sanctuary sites. These findings require validation through well-designed prospective multicenter trials to establish evidence-based management guidelines.

## ACKNOWLEDGMENTS

This work was supported by Anhui Provincial Department of Education Scientific Research Project (grant number 2023AH010079).

## ETHICAL APPROVAL

This study was approved by the Ethics Committee of the First Affiliated Hospital of the University of Science and Technology of China (2025-RE-007).

## AUTHOR CONTRIBUTIONS

X.Z. designed the study and revised the manuscript. B.Y., X.Z., and Y.W. collected the clinical data. Z.S. analyzed data and wrote the manuscript. All authors have reviewed and approved the final version of the manuscript.
